# A metabolomics study in aqueous humor discloses altered arginine metabolism in Parkinson’s disease

**DOI:** 10.1186/s12987-023-00494-5

**Published:** 2023-12-04

**Authors:** Joan Serrano-Marín, Silvia Marin, David Bernal-Casas, Alejandro Lillo, Marc González-Subías, Gemma Navarro, Marta Cascante, Juan Sánchez-Navés, Rafael Franco

**Affiliations:** 1 Department of Biochemistry and Molecular Biomedicine, Universitat de Barcelona, Barcelona, Spain; 2 Institute of Biomedicine of University of Barcelona (IBUB), University of Barcelona (UB), Barcelona, 08028 Spain; 3 CIBEREHD. Network Center for Hepatic and Digestive Diseases, National Spanish Health Institute Carlos III (ISCIII), Madrid, 28029 Spain; 4 Department of Genetics, Microbiology and Statistics, Faculty of Biology, Universitat de Barcelona (UB), Barcelona, 08028 Spain; 5 Department of Biochemistry and Physiology, Universitat de Barcelona, Barcelona, Spain; 6 CiberNed. Network Center for Biomedical Research in Neurodegenerative Diseases., Spanish National Health Institute Carlos iii, Av. Monforte de Lemos, 3-5, Madrid, 28029 Spain; 7 Department of Ophthalmology, Ophthalmedic and I.P.O. Institute of Ophthalmology, Palma de Mallorca, Spain; 8 School of Chemistry, Universitat de Barcelona, Barcelona, Spain

**Keywords:** Levodopa, Putrescine, Linear discrimination, Sensitivity, Biogenic amines, Carnitine, Eye, Spermidine, Mass spectrometry

## Abstract

**Background:**

The lack of accessible and informative biomarkers results in a delayed diagnosis of Parkinson’s disease (PD), whose symptoms appear when a significant number of dopaminergic neurons have already disappeared. The retina, a historically overlooked part of the central nervous system (CNS), has gained recent attention. It has been discovered that the composition of cerebrospinal fluid influences the aqueous humor composition through microfluidic circulation. In addition, alterations found in the brain of patients with PD have a correlate in the retina. This new paradigm highlights the potential of the aqueous humor as a sample for identifying differentially concentrated metabolites that could, eventually, become biomarkers if also found altered in blood or CSF of patients. In this research we aim at analyzing the composition of the aqueous humor from healthy controls and PD patients.

**Methods:**

A targeted metabolomics approach with concentration determination by mass spectrometry was used. Statistical methods including principal component analysis and linear discriminants were used to select differentially concentrated metabolites that allow distinguishing patients from controls.

**Results:**

In this first metabolomics study in the aqueous humor of PD patients, elevated levels of 16 compounds were found; molecules differentially concentrated grouped into biogenic amines, amino acids, and acylcarnitines. A biogenic amine, putrescine, alone could be a metabolite capable of differentiating between PD and control samples. The altered levels of the metabolites were correlated, suggesting that the elevations stem from a common mechanism involving arginine metabolism.

**Conclusions:**

A combination of three metabolites, putrescine, tyrosine, and carnitine was able to correctly classify healthy participants from PD patients. Altered metabolite levels suggest altered arginine metabolism. The pattern of metabolomic disturbances was not due to the levodopa-based dopamine replacement medication because one of the patients was not yet taking levodopa but a dopamine receptor agonist.

**Supplementary Information:**

The online version contains supplementary material available at 10.1186/s12987-023-00494-5.

## Introduction

Parkinson’s disease (PD) is a neurodegenerative disorder that primarily affects the motor control system. The molecular aspects of PD are complex and multifactorial, involving several processes that contribute to the degeneration of neurons in the substantia nigra of the human brain. A key pathophysiological feature is the accumulation of misfolded and aggregated proteins, such as alpha-synuclein in several regions of the central nervous system (CNS), including the hippocampus, the locus coeruleus, and the retina [1–4]. These protein aggregates form intracellular inclusions known as Lewy bodies, which are a hallmark pathological feature of the disease [5, 6]. Another important aspect of PD is oxidative stress, which results from an imbalance between the production of reactive oxygen species (ROS) and the antioxidant defense mechanisms in cells. This can lead to damage to cellular components, including proteins, lipids, and DNA. Ultimate the failure of movement control in patients is due to the severe reduction in dopamine production by nigral neurons.

Therapy for PD patients usually begins with dopamine replacement therapy that involves administering 3-(3,4-Dihydroxyphenyl)-L-alanine (levodopa, CAS 59-92-7), which is a dopamine precursor. Complications from chronic levodopa (L-DOPA) administration develop over time, eventually requiring surgery and deep brain stimulation [7–10]. Unfortunately, there is no effective therapy to slow the progression of the disease. Furthermore, there is a need to find biomarkers to assess the rate of disease progression and the efficacy of neuroprotective therapies.

Finding biomarkers of neurological disease in the blood is highly difficult mainly due to the complex fluid composition and the little influence of the CNS compared to that of the renal, respiratory and immune systems. Another possible approach is collecting cerebrospinal fluid (CSF); since it is in direct contact with the CNS it is expected to be more informative than the blood. Yet another interesting fluid is the aqueous humor (AH) due to the connection to the nervous system through microfluidic circulation and due to its intimate relationship with retina [11–14]. It should be noted that both the retina and the eye physiology are affected in many neurological diseases [3, 15]. Despite this knowledge, there has not been much use of ocular fluids to better understand how neurological disturbances are affecting ocular fluids/structures. It is known that although the AH needs to have an adequate refractive index (so its osmolarity must be limited), its composition is varied and contains several types of molecules [16–19].

Recent advances in metabolomic methods allow the determination of the level of tens of compounds in small amounts of biological samples. A previous report has shown the metabolome pattern of the serum of PD patients and has identified six potential biomarkers of the disease: 3-hydroxykynurenine, ornithine and four amino acids, homoserine, ß-alanine, aspartic acid and tyrosine [20]. The objective of this work was to establish the metabolome pattern in the AH of PD patients. Despite AH cannot become a fluid for use in diagnosis or assessment of disease progression, differences in the composition of AH from healthy controls and patients with PD could guide the discovery of biomarkers in blood and CSF. The concentration of 188 metabolites, from total sugars and lipids (lysophosphatidylcholines and sphingomyelins) to acyl-carnitines, biogenic amines and amino acids, was determined. Significant differences were found for amino acids, biogenic amines and, importantly, acylcarnitines. Concentration variation of the selected compounds was correlated, thus suggesting that changes were due to a common mechanism.

## Results

### Analysis of differentially concentrated molecules

**Fig. 1:**
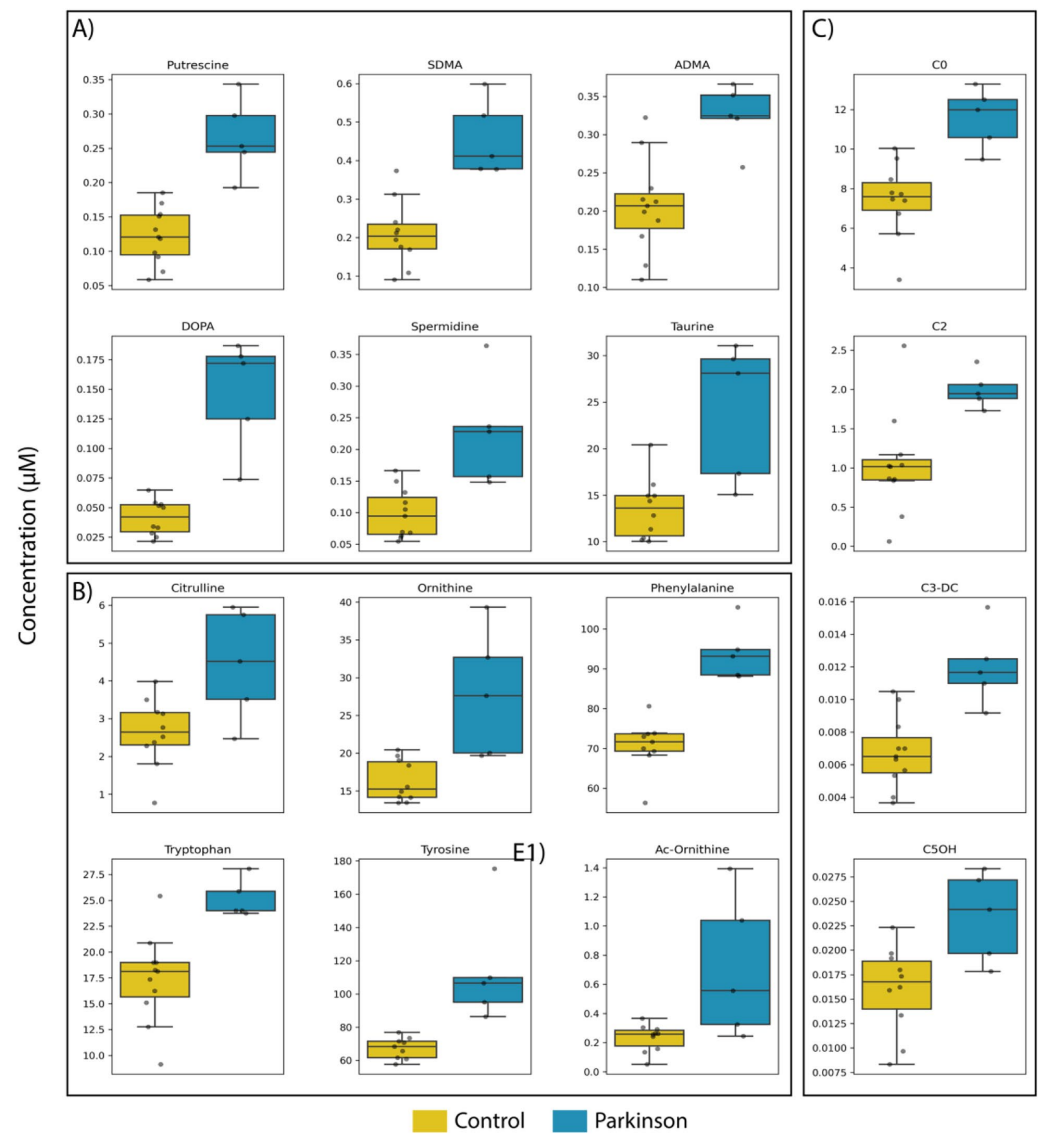
Univariate analysis of metabolites in the AH of the two groups. Median in box-and-whisker plots (whiskers indicate the furthest data points in lower and upper ranges determined by 1.5 times the interquartile range in the two groups, control and parkinsonian). **(A)** Biogenic amines. **(B)** Amino acids. **(C)** Acylcarnitines

Analysis of data from healthy controls and patients diagnosed with PD led to reliable concentrations for 72 compounds. Individual values of compounds with altered levels in the AH of PD samples are provided in Supplementary Table S1). Metabolites with concentration values below the detection limit or with concentration values that are not within the standard curve were not included in the analysis. With very few exceptions the level of metabolites was higher in the AH of PD patients. When comparing the data in samples from PD patients and healthy controls, the concentration of 16 compounds was significantly different (Supplementary Table S1). The greatest range of variation between patients and controls and the highest statistical score was obtained with L-DOPA, the dopamine precursor used in PD therapy (Supplementary Figure S1). An internal-like control is the amount of L-DOPA in a PD patient that has been not yet prescribed L-DOPA but pramipexole (CAS 104632-26-0), a dopamine receptor agonist [21, 22]. The level of the compound in the AH of this patient is within the range found in the control group (Supplementary Table S1). This fact explains the atypical value in the box-and-whisker patients’ plot of L-DOPA (blue) in Fig. 1A.

Metabolites differentially concentrated in the AH of PD patients were grouped into biogenic amines, amino acids and acylcarnitines. In addition to L-DOPA, the levels of five biogenic amines, putrescine, spermidine, taurine, symmetric dimethylarginine (SDMA), and asymmetric dimethylarginine (ADMA), were elevated in the AH of patients). C0, C2, C3-DC, and C5OH acylcarnitine levels were increased in the AH of the PD group. Finally, the differentially concentrated amino acids were citrulline, ornithine, acetyl-ornithine, phenylalanine, tryptophan, and tyrosine (Supplementary Figure S1).

Box-and-whisker plots for differentially elevated biogenic amines, other than L-DOPA, in the AH of patients show that discrimination between control and PD groups is possible using the levels of putrescine, SDMA or ADMA. Values for taurine and spermidine are more scattered with superposition of the whiskers of yellow and blue boxes in Fig. 1A.

Box-and-whisker plots for differentially elevated amino acids in the AH of patients show that discrimination between control and PD groups is possible using the levels of phenylalanine, tyrosine and tryptophan. For these 3 molecules the whiskers of the two groups were not overlapping. In addition, the concentration of these amino acids is high, in the micromolar range, i.e., their level can be efficiently and reliably measured by standard methods not requiring mass spectrometry. The median values of the other three amino acids are elevated in the PD group, but the individual values were quite variable from one individual to another.

Acylcarnitines are gaining relevance for presence in mitochondria, which is an organelle that is often dysfunctional in degenerating neurons. First, it should be noted that the method used allowed the measurement of several acyl carnitines in the AH. Box-and-whisker plots for differentially acylcarnitines in the AH of patients show that discrimination between control and PD groups is possible using just the level of C2 acylcarnitine. Unlike C3-DC and C5OH, the level of the C2 acylcarnitine in the AH is in the micromolar range, indicating that it can be easily measured without the need to use mass spectrometry.

### Receiver operating characteristic (ROC) analyses to examine the diagnostic ability of each of the significant metabolites

**Fig. 2:**
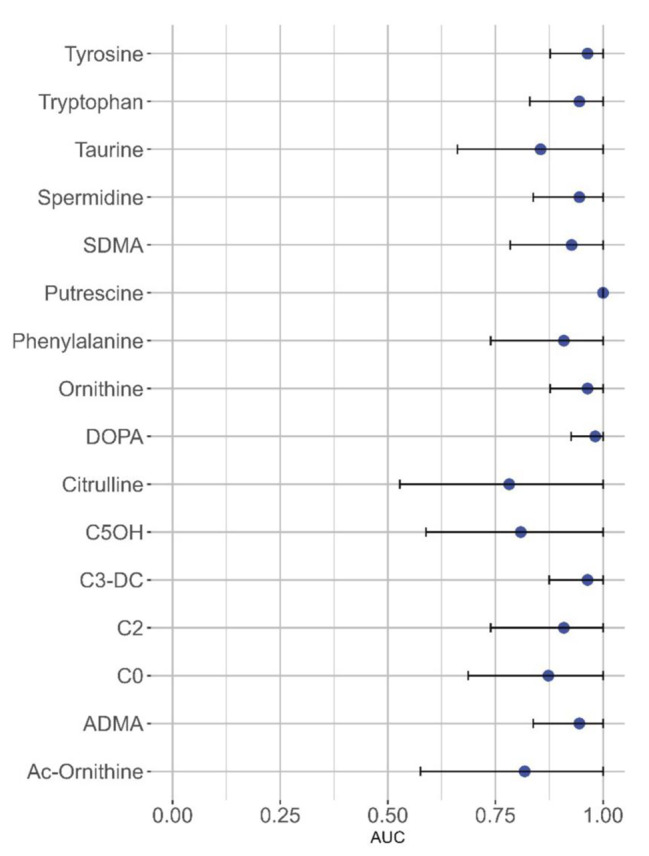
Forest plot showing confidence intervals at the 95% level of the AUC of the differentially concentrated metabolites. The minimal and maximal interval values are in the last two columns of Table 1

After dissecting the three families of metabolites, we evaluated the predictive power of the level of each of the 16 metabolites (individually) by calculating its sensitivity and specificity in discriminating controls and PD patients. The receiver operating characteristic (ROC) curves for each of the biogenic amines depicted in Fig. 1A is shown in supplementary Figure S2A, while the specific parameters are shown in Table 1. Due to the stringent criteria of selection of differentially elevated molecules sensitivity and specificity values were high for all biogenic amines. The area under the ROC (AUC) for the putrescine was 1 and also the sensitivity and the specificity were 1. The cutoff value, 0.189 µM, is high enough to be measured by standard clinical chemistry techniques. Sensitivity was also 1 for the remaining biogenic amines for which specificity varied between 0.73 for taurine and 0.91 for L-DOPA or SMDA. Therefore, the cutoff values for all these compounds seem reliable. The AUC for each metabolite and its 95% confidence interval is displayed in a Forest plot (Fig. 2). Interestingly, putrescine is not only the most discriminative metabolite in terms of sensitivity and sensitivity but it also has the smallest confidence interval. Therefore, a single parameter, putrescine concentration, even with a relatively small number of samples, is robust enough to identify those AH coming from PD patients.

The ROC curves for each of the amino acids included in Fig. 1B is shown in supplementary Figure S2B, while the specific parameters are shown in Table 1. Except for citrulline and Ac-ornithine, the sensitivity for the amino acids that were differentially elevated in the PD group was 1. Specificity was notable, 0.91, for Trp, Tyr, Phe and ornithine, with cutoff values that were relatively high, ranging from 19.7 µM in the case of ornithine to a remarkable 81.6 µM in the case of Tyr.

Finally, the ROC curves for each of the acylcarnitines depicted in Fig. 1C is shown in supplementary Figure S2C, while the specific parameters are shown in Table 1. In all cases the sensitivity was 1 while specificity varied from a modest 0.55 (C5OH) to a notable 0.91 (C2). The C2 is the acylcarnitine that shows a significant increase in PD combined with a cutoff value in the micromolar range, maximal sensitivity, and elevated specificity, meaning that this acylcarnitine in combination with an amino acid with high scores and/or a biogenic amine with high scores could be used to detect whether a given AH is from a patient or a control.

Confidence intervals are shown in the last two columns of Table 1 and in Fig. 2. Due to the high variability when evaluating molecules in human samples, wide confidence intervals were expected. However, confidence intervals for tyrosine. Tryptophan, ornithine C3-DC acylcarnitine, ADMA were relatively small. Obviously, the confidence interval for the precursor of dopamine, L-DOPA, which is used in PD therapy, displayed a small confidence interval. Furthermore, putrescine stands out for having a very low degree of uncertainty. The striking discriminative performance of putrescine requires re-testing in a large cohort to be confirmed.

**Table 1:** Maximum values of sensitivity and specificity and area under the curve in the ROC plots shown Supplementary Figures S2. The AUC, cutoff (µM), the lower and upper limits (95% confidence interval) are indicated

	Sensitivity	Specificity	AUC^a^	Cutoff (µM)	Lower limit	Upper limit
C0	1	0.727	0.873	8.975	0.69	1.0
C2	1	0.909	0.909	1.664	0.74	1.0
C3-DC	1	0.818	0.964	0.009	0.87	1.0
C5OH	1	0.545	0.809	0.018	0.59	1.0
Cit	0.8	0.818	0.782	3.507	0.53	1.0
Orn	1	0.909	0.964	19.658	0.88	1.0
Phe	1	0.909	0.909	84.417	0.74	1.0
Trp	1	0.909	0.945	22.308	0.83	1.0
Tyr	1	0.909	0.964	81.583	0.88	1.0
AcOrn	0.8	0.818	0.818	0.314	0.58	1.0
ADMA	1	0.818	0.945	0.244	0.84	1.0
L-DOPA	1	0.909	0.982	0.069	0.93	1.0
Putrescine	1	1	1.000	0.189	1.0	1.0
SDMA	1	0.909	0.927	0.375	0.78	1.0
Spermidine	1	0.818	0.945	0.140	0.85	1.0
Taurine	1	0.727	0.855	14.992	0.66	1.0

^a^AUC, Area under the curve

### Unsupervised analysis for discriminating PD from control cases

Above the level of each metabolite was analyzed individually, however, some of the data are likely to be correlated, that is, variation in the concentration of one metabolite may occur concomitantly with variation in the concentration of other metabolites. For such complex scenarios, we have used algorithms to reveal interactions/correlations that can result in patterns. One of them is the principal component analysis (PCA). Very briefly, PCA aims to detect the correlation between variables and to identify patterns in the data.

**Fig. 3:**
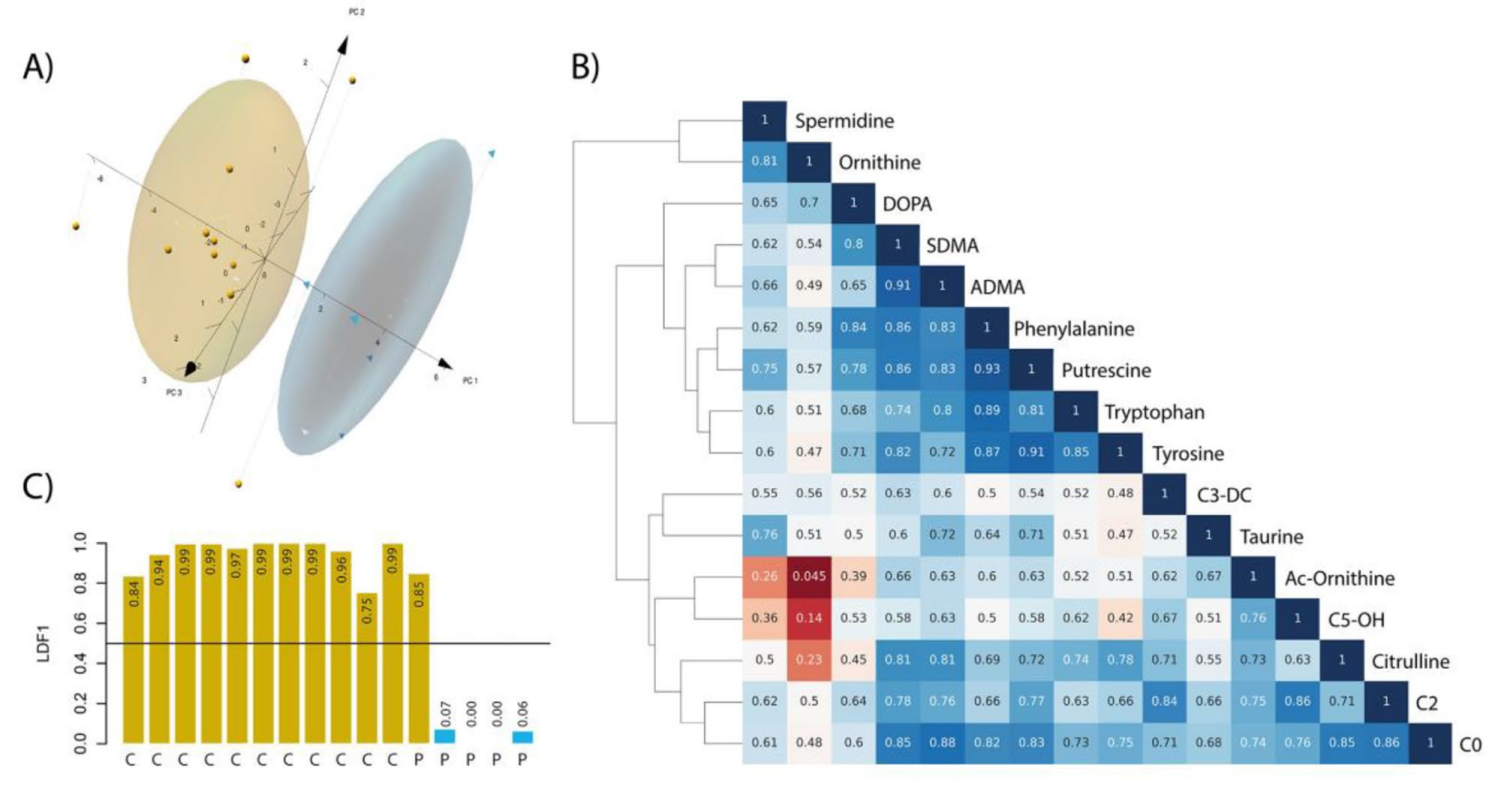
Multivariant metabolite analyses. **(A) Principal component analysis diagram.** The space and data for controls are in yellow and the space and data for PD individuals are in blue. Principal component (PC) 1, PC2, and PC3 explain, respectively, the 63.8%, the 12.0%, and the 9.0% of the between-group variance. **(B) Dendrogram and heatmap**. It shows the similarity index between the differentially concentrated metabolites in PD patients. The similarity index was obtained using Pearson’s correlation coefficient (the darker the blue, the higher the similarity index). Compounds displaying a similarity index greater than 0.5 are in blue the darker the red, the lower the similarity index). **(C) Linear discrimination analysis.** Probability of being classified as a control using the LDF1 function with cross-validation. The colors of the bars indicate the classification result (gold: control (C); blue: PD patient (P)). The overall performance of the cross-validated function is 93.75%

By applying PCA (excluding L-DOPA), we observed a good segregation between controls and PD patients, making possible to distinguish individuals with very few false negatives and few false positives. Figure 3 A shows the PCA diagram; The principal component (PC) 1, the PC2, and the PC3 explain, respectively, the 59.1%, the 12.7%, and the 9.9% of between-group variance.

### Supervised analysis for discriminating PD from control cases

Given the performance of PCA, we were looking for the correlation values between the 16 significant molecules with the goal of developing a supervised learning algorithm. Pearson’s coefficient calculation led to the image of Fig. 3B. The dendrogram shows a strong dependence among parameters. It is worth highlighting the high number of Pearson correlation coefficients greater than 0.5. It is noteworthy that putrescine appears with strong correlation with all other metabolites that are differentially concentrated; particularly high were the correlations between putrescine and phenylalanine (0.93) and between putrescine and tyrosine (0.91). Interesting was the apparently contradictory finding of the poor correlation between ornithine and Ac-ornithine.

As above mentioned, the measurement of the level of putrescine would serve to distinguish among controls and PD in the AH samples here used. To estimate how precise a predictive model would be in practice, i.e., in a higher number of samples, a supervised linear discrimination analysis (LDA) with leave-one-out cross-validation was performed using three metabolites, one from each group of compounds, biogenic amines, amino acids, and acylcarnitines.

We selected three metabolites considering their role in different metabolic pathways and their discriminative capacity: putrescine, tryptophan and C0 acylcarnitine. To minimize correlation bias, we selected a compound from each category: biogenic amines, amino acids and acylcarnitines. Putrescine is the most correlated metabolite as shown in Fig. 3B. Tyrosine was replaced by tryptophan because tyrosine is related to dopamine metabolism and may be affected by L-DOPA therapy. Assuming an interplay between correlation and significance to enhance discriminative power, the discriminant function using the linear LDA approach was.

LDF1 (Y)= -4.02 + 22.00 [Putrescine] + 0.15 ([Trp]/[C0])

Where Trp is tryptophan, and C0 is carnitine itself.

Using this equation, the degree of discrimination is greatest when classifying the control group but misclassifying one of the five patients; the overall model performance using the discriminant function is 93.7%. In any case LDA analysis shows good assignment to control or PD group considering the concentration of just three metabolites. The probability of proper classification for all cases (11 controls and 5 patients) using putrescine, Trp and carnitine after cross-validation (leave-one-out) is in Fig. 3C.

### Pathway enrichment analysis

**Fig. 4:**
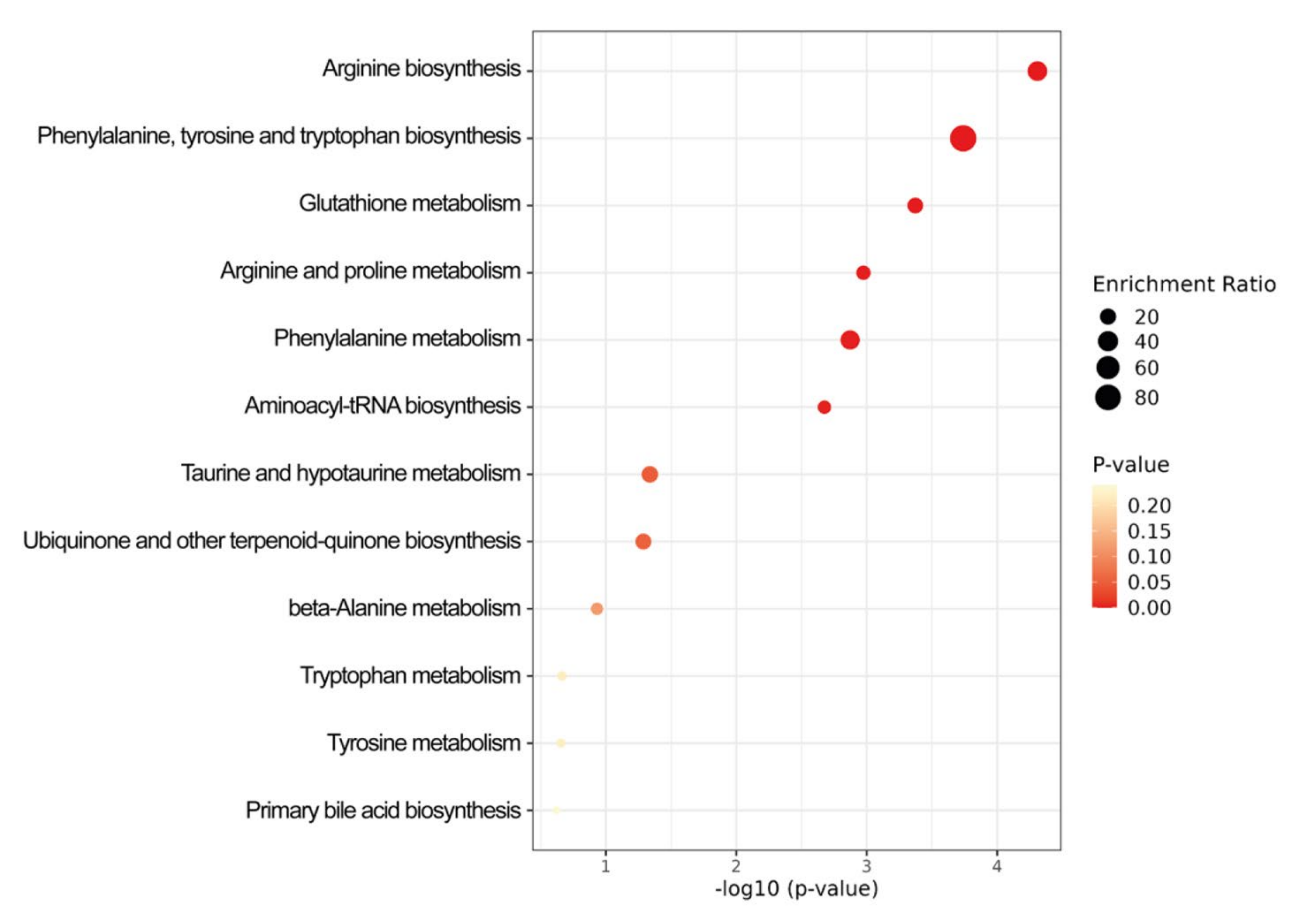
Metabolic pathways in which differentially concentrated compounds are involved. The diameter of every point red indicates the enrichment ratio (value in PD versus value in control). The greater the reddish color, the lower the p-value. The enrichment ratio is calculated as the ratio between the number of metabolites in the dataset that are involved in a specific metabolic pathway and the total number of metabolites associated with that pathway; the p-value is obtained using the hypergeometric test

To understand the level of general affectation within a biochemical framework, we performed a pathway enrichment analysis. Figure 4 shows metabolic pathways that are overrepresented in the AH of PD patients depicting both the degree of significance and the ratio of enrichment in each pathway. As one would expect from the nature of the differentially concentrated compounds, the metabolomics data here presented indicate alteration in the metabolism of amino acids, glutathione metabolism and in ubiquinone and aminoacyl-tRNA biosynthesis (Fig. 4). Remarkably, the most relevant pathway considering both enrichment ratio and p value is arginine biosynthesis.

## Discussion

Ocular fluids hold potential for enhancing our understanding of the pathophysiology of neurological diseases. This is because of their close association with the retina — a traditionally underestimated component of the central nervous system — and their connection with the CSF through microfluidic circulation [11, 14]. Despite metabolomics show promise in PD [23], to our knowledge the AH from PD patients, whose availability is very limited, has not been used in metabolomic studies. Alterations in the concentrations of compounds in AH can provide relevant information regarding pathophysiological mechanisms and understand whether a certain comorbidity (e.g. dry eye, retinal alterations) is the consequence of a chronic disease such as PD. Dopamine regulates retinal physiology and its lack thereof in PD may lead to serious and diverse types of ocular impairment [24, 25]. It should be noted that the patients in this study did not present any alteration of the retina, therefore, the variation in the composition of AH here reported is not caused by ocular degeneration.

Biomarkers for neurodegenerative diseases have been based more on genomic and proteomic techniques than on metabolomics. Genomics has made it possible to identify genes that can explain some of the cases of PD, those that are heritable [26–28]. However, most cases are idiopathic and, since they share the presence of α-synuclein aggregates, it has been considered that this protein could be a biomarker. By mechanisms not fully established, but which could include transmission by exosomes, α-synuclein can appear in plasma and in cerebrospinal fluid (CSF). At present, the determination of the concentration of α-synuclein in plasma or CSF is not considered to be a fully reliable biomarker, that is, complementary biomarkers are required [29–31]. Metabolomic approaches offer promising perspectives that, even though in a nascent stage, . One of the objectives is early diagnosis in the asymptomatic phase of PD and another is monitoring the progression of the disease. We are aware that the AH cannot be used for diagnosis because it requires surgery to get it and that the number of AH samples from patients is a limitation of our study. Our approach has been to use this unique material obtained from PD patients, to look for information that could be useful to know better the metabolic consequences of the disease but also to detect molecules that could be differentially expressed also in blood and/or CSF and then become biomarkers of the disease. Our results point to biogenic amines as candidates to be differentially expressed in blood and CSF and if so, become biomarkers. It is important the concept also arising in the present paper that classification of control versus cases may be done using the concentration of a single metabolite or considering the concentration of 2 to 4 metabolites. It would also be very valuable to evaluate, in blood and/or CSF and, if possible, in HA, if the differentially concentrated metabolites vary in qualitative and/or quantitative terms depending on age, sex and, very relevant, genotype (in the case of familial PD).

Alterations in putrescine levels correlate with certain types of biological stress, particularly those with a significant oxidative stress burden. Its role is relevant not only in mammals [32, 33], but also in other species, including bacteria [34] and plants [35], suggesting a common ancestral system for maintaining REDOX homeostasis. The metabolomics study by Klatt et al. does not find variation of the level of this molecule in serum of PD patients [20]. Putrescine level variations in plasma can occur in various other types of disorders not related to the nervous system [36, 37], but it is decreased in the serum of Alzheimer’s disease patients [38]. Therefore, the increase in the AH of PD patients with a maximal sensitivity and maximal specificity is remarkable, especially considering that the other variations here identified correlate with the variations of putrescine. Putrescine has been reported to have a protective effect against apoptosis caused by mitochondrial dysfunction [39]. Therefore, its increased concentration could be attributed to both, an upregulation in its synthesis and/or an increase in cellular death, leading to the release of intracellular metabolites into the extracellular matrix.

Putrescine levels in the AH of PD patients exhibits a positive correlation with the concentration of the other 15 metabolites that display differential levels, thus, suggesting that putrescine has interactions within the metabolic networks in which these other metabolites are involved. Ornithine, which is the precursor of polyamines, putrescine included, was also increased in the AH of PD patients. Remarkably, ornithine but not putrescine is elevated in the serum of PD patients; however, ornithine is slightly decreased in the CSF of PD patients [20, 40–43], indicating that that exploring novel biological fluids may help to better understand PD pathophysiology and disease progression.

There are several other relevant differences when comparing metabolomics results in serum or CSF and AH. For instance, those concerning the pattern of differentially elevated amino acids; in serum there are four that significantly increase in PD samples: homoserine, ß-alanine, aspartic acid and tyrosine, whereas in AH tyrosine increases but not the other three. In addition, the AH of patients presents significant increases in the concentration of phenylalanine, citrulline, acetyl-ornithine, ornithine, and tryptophan. In the case of tryptophan, its level increases in AH while it decreases in the serum of patients. In the CSF of patients there is not any increase in the level of amino acids, except for taurine [40]. In summary, the pattern of amino acid level variation in the AH of PD patients is markedly different than that described in both plasma and CSF. In addition, the increase of dimethylarginines detected in the AH of PD patients is not found in the CSF of patients [44] proving that the pattern of metabolomic variation in PD is unique in AH, that is, it is different from that reported in serum and CSF.

Interestingly, when comparing the results concerning amino acids with data obtained in CSF of PD patients [45–47], there are both similarities and differences. The concentration of tyrosine, putrescine, citrulline, ornithine and phenylalanine were increased in both AH and CSF of patients. In contrast, taurine and spermidine were decreased in CSF while increased in AH. We did not find significant changes in the concentration of leucine, isoleucine, valine and alanine that are reportedly increased in the CSF of PD patients. Based on our results and on the potential involvement of mitochondrial alterations in nigral neuronal death, we consider it very relevant to evaluate the concentration of acylcarnitines in the CSF of patients.

**Fig. 5:**
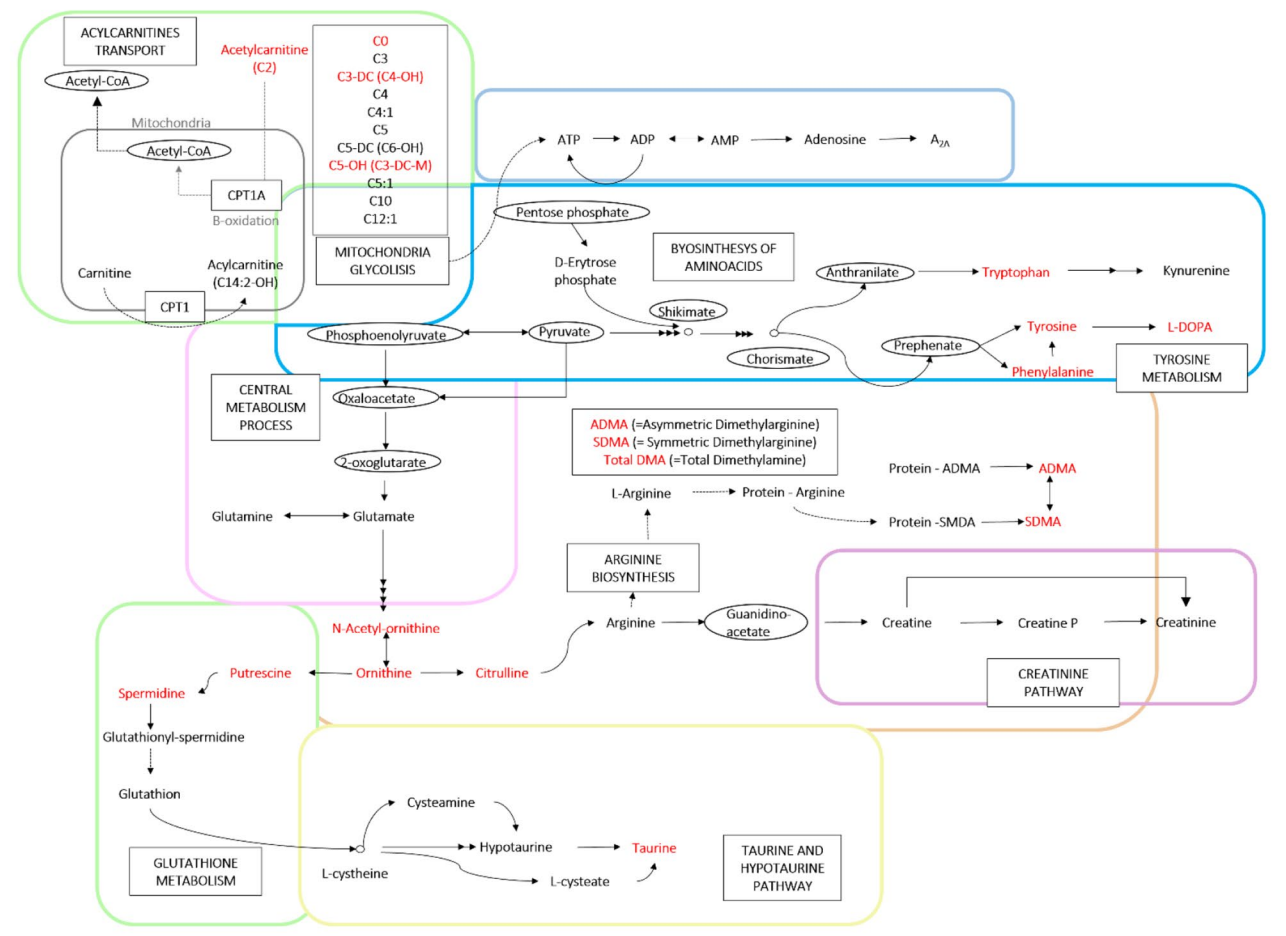
Metabolic pathway map summarizing the results. The colored boxes indicate the different metabolic processes included in the scheme. Metabolites differentially concentrated in PD samples are in red

High-throughput metabolomics makes it possible to provide information on the metabolisms that are altered in PD. Considering the data in AH, there was an alteration in the level of free carnitine and of various acylcarnitines. This result fits with the notion that mitochondria of degenerating neurons are altered, and carnitines are key to facilitate the transport of lipids through mitochondrial membranes (see [48–50]). This assumption is reinforced by the altered acylcarnitine levels found in the serum of PD patients where long-chain acylcarnitine levels are decreased [51]. Our data and those reported in plasma [20] coincide in alterations in the tryptophan/kynurenine pathway and in the metabolism of polyamines. AH data also point to important alterations in arginine metabolism. There are five metabolites the levels of which have been found to be altered in the AH of patients and participate in arginine metabolism: citrulline, N-acetylornithine, ADMA, SMDA and total DMA (Fig. 5). The significant correlation between putrescine and all the other 15 differentially concentrated metabolites suggests that PD alterations may be influencing a common regulator at a higher hierarchical level, such as a gene or a limiting enzyme. Identifying this shared regulator could offer valuable insights into the pathophysiology of PD.

Given its potential to stimulate growth hormone, exogenous arginine administration has been used for the differential diagnosis of PD [52]. However, the metabolism of arginine is not directly associated with that of catecholamines in general and of dopamine in particular. Therefore, not much attention has been paid to how arginine and its metabolism products may be affected in PD and/or may impact neurodegeneration mechanisms. One possibility that should be explored is whether the alterations in arginine levels are related to alterations in the production of nitric oxide, which is derived from the amino acid. Indeed, there is evidence that the plasticity derived from neurodegeneration and medication (L-DOPA in PD) is mediated, at least in part, by nitric oxide (recent reviews: [53, 54]).

The presence of N-acetylornithine in AH is intriguing because the enzymes that specifically handle this compound are not expressed in mammals. N-acetylornithine carbamoyltransferase (E.C. 2.1.3.9) uses the compound to synthesize N-acetyl-L-citrulline, which is then processed to arginine. Additionally, glutamate N-acetyltransferase (E.C. 2.3.1.35) catalyzes the interconversion of glutamate and N-acetyl-L-citrulline to L-ornithine and N-acetyl-glutamate.

To our knowledge, the role of N-acetylornithine in mammals is not considered relevant; the mammalian enzyme putatively using N-acetylornithine is NAT8, a member of the “NAT” acetyl transferase family of enzymes. NAT8 and/or NAT8 homologues are found in the genome of all vertebrate animals and appear to be related to the metabolism of xenobiotics [55]. In contrast, N-acetylornithine is relevant in the synthesis of arginine in microorganisms [56] and of ornithine in plants [57]. N-acetylornithine carbamoyltransferase (E.C. 2.1.3.9) may use N-acetylornithine to synthesize N-acetyl-L-citrulline, which can be either processed to arginine or to L-ornithine and N-acetyl-glutamate upon acetyl exchange catalyzed by glutamate N-acetyltransferase (E.C. 2.3.1.35). It is intriguing that no mammalian enzyme is reportedly able specifically handle N-acetylornithine. In the absence of a specific mammalian enzyme the increase of N-acetylornithine may be due to a side reaction (not catalyzed by any enzyme) or is consequence of reactions in microbiota. The last possibility could fit with the increasing evidence on the role of microbiota in diseases affecting the CNS [58–61].

## Methods

### Subjects

A total of 16 samples were collected; 11 samples of healthy controls (3 males and 8 females, mean age 42.9, range 24–66, and 5 from PD patients (all 5 were male, mean age 72.4, range 69–79) were collected. None of the patients or healthy controls reported any kidney disease, and creatinine and urea plasma levels were within reference values. Axial length (AXL) was 23.92 mm (21.25–27.29 range) in the control group and 24.37 mm (23.23–25.32 range) in the PD group. None of the individuals had previously undergone ocular surgery. One PD patient was in stage two and was not yet on L-DOPA treatment. The other patients were in stage 4, under treatment with L-DOPA and with an average of 5.3 years since the first diagnosis, made according to criteria in Postuma. et al., (2015) [62]. AH samples (100–200 µl) were collected aseptically by paracentesis and avoiding contact with the iris or blood vessels. The humor was obtained during the first step of surgery, following administration of an anesthetic drop over the eye surface (2% lidocaine); a 1 mm corneal microincision was made while visualizing the eye though a surgical microscope. A 30 gauge Rycroft cannula attached to a tuberculin syringe was used to aspirate the sample. After the surgery samples were immediately placed at -20 ºC and stored at this temperature until processing.

Participants were informed and, in terms of ethical standards, this study adhered to the tenets of the declaration of Helsinki. The study has been evaluated by the *Comitè d’Ètica de la Investigació de les Illes Balears (CEI-IB)*; the committee deemed the study exempt from review. All but one PD patients were under L-DOPA therapy; the patient that had not yet taken L-DOPA was under pramipexole therapy. None of them showed any sign of retinopathy. The AH was collected at the beginning of the intervention, in both PD and control groups there were individuals with myopia or hyperopia but without cataract or eye pathologies; they underwent surgery for refractive lensectomy or ICL implanting (without withdrawing the crystalline lens).

Surgeries were performed in fasting conditions by JS-N. Pupil was dilated and disinfected with 5% povidone iodine 5. Topical anesthesia was used, and the surgery protocol was identical in all cases. The first side port, approximately, 1 mm width, was performed using the microscope in the operating room. 100–150 µl AH was aspirated with a 27G needle. Samples were immediately transferred to 0.5 ml Eppendorf tubes and stored at -80ºC until analysis.

### Metabolomics

The AbsoluteIDQ™ p180 Kit (Biocrates Life Sciences, Innsbruck, Austria), was used. 188 metabolites, from biogenic amines, amino acids, hexoses, phospho- and sphingolipids and acyl-carnitines can be determined using this specific kit. Individual metabolites may be found in www.biocrates.com/products/research-products/absoluteidq-p180-kit. The inter-laboratory reproducibility of the platform using human plasma/serum and details of the associated methodology have been reported elsewhere; using standard reference (human) plasma the reported median precision and inter-laboratory accuracy of the assay were, respectively, 6.7% and 107% [63].

Sample processing was as indicated by the manufacturer. Briefly, 10 µl of internal standard (provided by the kit) as well as 10 µl of Phosphate buffered saline (PBS) (used as blank), 10 µl of standards, 10 µl of quality controls (provided by the kit), or up to 30 µl of AH were plated in each well. Samples were dried down at room temperature under nitrogen gas flow. Next, phenylisothiocyanate (PITC) solution (PITC 5% V/V in ethanol:water:pyridine 1:1:1 solvent) was added to each well. After 20 min incubation at room temperature, all the wells were dried down under nitrogen gas flow. Metabolites were then resuspended in 300 µl of extraction solvent (5 mM ammonium acetate in methanol). For the analysis, samples were diluted 1:1 with milliQ water (for tandem mass spectrometry coupled to high pressure liquid chromatography (HPLC/MS/MS)) or diluted 1:10 with FIA mobile phase (flow injection analysis (FIA) coupled to MS/MS). The analyses were performed in the AB Sciex 6500 QTRAP MS/MS mass spectrometer (AB Sciex LLC, Framinghan, MA, USA) coupled to an Agilent 1290 Infinity ultra-high pressure liquid chromatography (UHPLC) system (Agilent, Santa Clara, CA, USA).

HPLC/MS/MS settings: HPLC column was Agilent ZORBAX Eclipse XDB C18, 3.0 × 100 mm, 3.5 μm (Agilent, Santa Clara, CA, USA). Column oven was set up at 50 ºC. HPLC mobile phases were water + 0.2% formic acid (solvent A) and acetonitrile + 0.2% formic acid (solvent B). Flow rate was maintained at 0.5 ml/min for all the analysis. HPLC gradient started with 0.5 min at 100% of solvent A, then a gradient for 5 min up to 95% of solvent B, then 1 min at 95% of solvent B, and then a gradient for 0.5 min up to 100% of solvent A. These final conditions were maintained for 2.5 min. Ionization source of MS/MS was electrospray ionization (ESI) in positive mode. ESI probe position was x-axis = 8 and y-axis = 0. Data was recorded using Multiple Reaction Monitoring (MRM) scan type, using the methods provided by the kit.

FIA/MS/MS settings: FIA mobile phase was methanol + FIA Mobile phase Additive provided by the kit. FIA gradient started at a flow rate of 0.03 ml/min for 1.6 min, then flow increased linearly until reaching 0.20 ml/min after 0.8 min. This flow was maintained for 0.4 min. Finally, flow decreased up to 0.03 ml/min in 0.2 min. Ionization source of MS/MS was ESI in positive mode. ESI probe position was x-axis = 5 and y-axis = 5. Data was recorded using MRM scan type, using the methods provided by the kit.

Data analysis was performed using the Analyst (AB Sciex LLC, Framinghan, MA, USA) and the MetIDQ™ (Biocrates, Life Sciences, Innsbruck, Austria) software. Concentrations calculated were corrected considering the real volume of sample plated in each well.

### Statistical analysis

Outliers identified with the ± 2SD criterion were removed from statistical analysis. Univariate analysis of the data was performed using the two-sample t-test comparing one by one all compounds with reliably determined levels. . Differences were considered significant when the adjusted p value was < 0.05. The Python code used for these operations is uploaded to the GitHub page (https://github.com/JoanSerranoMarin/PD). Receiver operating ROC curves were performed using IBM SPSS software. Principal Component Analysis (PCA) was performed using R software.

We used Statgraphics 18 software to conduct a Linear Discriminant Analysis (LDA). We excluded DOPA due to its high dependence on medication intake. After standardizing the values, we proceeded with linear discriminant analysis using two approaches: one considering all metabolites and another employing a direct selection approach to identify the metabolites that best allow discrimination between PD patients and controls. The standardization method was based on the in-built R function scale, which centralizes the values by subtracting the average value and putting them on the same scale dividing by the standard deviation of each variable. To cross-validate using the leave-one-out approach, we used each data point as an individual test set, while training the model on all other data points. Consequently, at each iteration, a different data point was retained for testing purposes, while the model was tested with the remaining data. The performance of the linear discriminant function was subsequently evaluated based on these iterations. The total number of iterations was 15, which corresponds to the number of data points within the complete data set, after excluding, the reasons mentioned above. Cross validation was performed with R 4.3.0 software.

### Electronic supplementary material

Below is the link to the electronic supplementary material.

Supplementary Material 1. Figure S1: Volcano plot. Figure S2. ROC curves; Table S1: metabolite concentrations for every sample.

## Data Availability

The data produced in the study are in the published manuscript and/or in the Supplementary material. Source codes are available from the corresponding author upon reasonable request.

## Abbreviations

ADMA: Asymmetric dimethylarginine
AH: Aqueous Humor
CSF: Cerebrospinal fluid
LDA: Linear Discriminant analysis
PCA: Principal Component Analysis
PD: Parkinson’s disease
ROC: Receiver operating characteristic
SDMA: Symmetric dimethylarginine
