# Supplementary material for: A metabolomics study in aqueous humor discloses altered arginine metabolism in Parkinson’s disease
Source: Fluids Barriers CNS. 2023 Dec 4;20:90. doi: 10.1186/s12987-023-00494-5 (PMC10696737; doi:10.1186/s12987-023-00494-5)
Supplement: Supplementary file 1 — Supplementary Material 1. Figure S1: Volcano plot. Figure S2. ROC curves; Table S1: metabolite concentrations for every sample. [file 12987_2023_494_MOESM1_ESM.docx]

SUPPLEMENTARY MATERIAL

A metabolomics study in aqueous humor discloses altered arginine metabolism in Parkinson’s disease

Joan Serrano-Marín ^1,†^, Silvia Marin ^1,2,3,†^, David Bernal-Casas^4^, Alejandro Lillo ^5^, Marc González-Subías^1^, Gemma Navarro ^5,6^, Marta Cascante ^1,2,3^, Juan Sánchez-Navés ^7,‡^ and Rafael Franco ^1,6,8,‡,^*

^1^ Department of Biochemistry and Molecular Biomedicine. Universitat de Barcelona. Barcelona. Spain.

^2^ Institute of Biomedicine of University of Barcelona (IBUB), University of Barcelona (UB), 08028 Barcelona, Spain

^3^ CIBEREHD. Network Center for Hepatic and Digestive Diseases. National Spanish Health Institute Carlos III (ISCIII), 28029 Madrid, Spain

^5^ Department of Biochemistry and Physiology. Universitat de Barcelona. Barcelona. Spain.

^6^ Network Center for Biomedical Research in Neurodegenerative Diseases. CiberNed. Spanish National Health Institute Carlos iii. Av. Monforte de Lemos, 3-5. 28029 Madrid. Spain.

^4^ Department of Genetics, Microbiology and Statistics. Faculty of Biology, Universitat de Barcelona (UB). 08028 Barcelona, Spain. bernalcd@ub.edu

^7^ Department of Ophthalmology, Ophthalmedic and I.P.O. Institute of Ophthalmology, Palma de Mallorca, Spain.

^8^ School of Chemistry. Universitat de Barcelona. Barcelona. Spain.

† These authors contributed equally to this work.

^‡^ These authors contributed equally to this work.

***** Correspondence: [rfranco123@gmail.com](mailto:rfranco123@gmail.com)

**Supplementary Table S1. The individual concentration values (in μM) for healthy controls (C) and PD patients (P) are presented.**

**Blank cells correspond to data identified as outliers.**

| **Metabolite** | **C** | **C** | **C** | **C** | **C** | **C** | **C** | **C** | **C** | **C** | **C** | **P** | **P** | **P** | **P** | **P** | **adjp.value** | **Average_control** | **Average_Parkinson** | **FC** |
| --- | --- | --- | --- | --- | --- | --- | --- | --- | --- | --- | --- | --- | --- | --- | --- | --- | --- | --- | --- | --- |
| Phe | 71.67 | 80.67 | 73.00 | 73.67 |  | 68.33 |  | 70.00 | 73.83 | 69.33 | 56.36 | 88.50 | 88.17 | 94.83 | 105.50 | 93.17 | 0.001 | 70.76 | 94.03 | 1.33 |
| DOPA | 0.05 | 0.06 | 0.05 |  | 0.03 | 0.05 | 0.03 | 0.03 | 0.05 | 0.02 | 0.03 | 0.19 | 0.12 | 0.18 | 0.17 | 0.07 | 0.001 | 0.04 | 0.15 | 3.54 |
| Putrescine | 0.17 | 0.15 | 0.12 | 0.12 | 0.13 | 0.10 | 0.07 | 0.09 | 0.15 | 0.19 | 0.06 | 0.19 | 0.24 | 0.30 | 0.34 | 0.25 | 0.001 | 0.12 | 0.27 | 2.17 |
| C2 | 1.03 | 0.06 | 1.17 | 0.84 | 0.86 | 1.04 | 0.38 | 1.02 |  | 1.60 | 0.85 | 1.88 | 1.73 | 2.06 | 1.95 | 2.36 | 0.002 | 0.88 | 1.99 | 2.25 |
| SDMA | 0.19 | 0.18 | 0.17 | 0.21 |  | 0.37 | 0.11 | 0.24 | 0.31 | 0.22 | 0.09 | 0.38 | 0.38 | 0.52 | 0.60 | 0.41 | 0.003 | 0.21 | 0.46 | 2.18 |
| total DMA | 0.36 | 0.32 | 0.29 | 0.34 |  | 0.52 | 0.19 | 0.41 | 0.53 | 0.39 | 0.16 | 0.56 | 0.59 | 0.78 | 0.90 | 0.64 | 0.003 | 0.35 | 0.69 | 1.98 |
| C3-DC (C4-OH) | 0.01 | 0.00 | 0.01 | 0.01 | 0.01 | 0.01 | 0.00 | 0.01 | 0.01 | 0.01 | 0.01 | 0.01 | 0.01 | 0.01 | 0.01 | 0.02 | 0.007 | 0.01 | 0.01 | 1.78 |
| C0 | 6.74 | 7.40 | 7.73 | 7.47 | 7.80 | 8.47 | 3.40 | 9.53 |  | 10.03 | 5.73 | 9.48 | 10.58 | 11.98 | 12.50 | 13.28 | 0.008 | 7.43 | 11.57 | 1.56 |
| Spermidine | 0.09 | 0.11 | 0.06 | 0.13 | 0.07 | 0.06 | 0.05 | 0.07 | 0.15 | 0.17 | 0.12 | 0.16 | 0.36 | 0.23 | 0.24 | 0.15 | 0.008 | 0.10 | 0.23 | 2.31 |
| Orn | 14.22 | 20.43 | 14.13 | 13.43 | 13.47 | 19.03 |  | 14.93 | 18.38 | 19.65 | 15.55 | 32.70 | 39.33 | 19.67 | 27.62 | 20.03 | 0.009 | 16.32 | 27.87 | 1.71 |
| Trp | 18.98 | 20.87 | 18.13 | 15.10 | 25.43 | 12.77 | 9.13 | 16.23 | 18.98 | 18.25 | 17.36 | 23.75 | 24.02 | 25.87 | 28.07 | 24.00 | 0.009 | 17.39 | 25.14 | 1.45 |
| Tyr | 73.44 | 68.33 | 70.67 | 65.67 |  | 57.67 |  | 61.67 | 71.50 | 76.83 | 60.91 | 86.33 | 95.00 | 109.83 | 175.33 | 106.50 | 0.009 | 67.41 | 114.60 | 1.70 |
| ADMA | 0.21 | 0.20 | 0.17 | 0.19 | 0.29 | 0.23 | 0.11 | 0.21 | 0.32 | 0.22 | 0.13 | 0.26 | 0.32 | 0.37 | 0.35 | 0.32 | 0.009 | 0.21 | 0.32 | 1.57 |
| Taurine | 10.17 | 14.93 | 10.40 | 16.13 | 14.37 | 12.83 | 14.93 | 11.33 |  | 20.38 | 10.05 | 15.05 | 29.62 | 31.05 | 17.32 | 28.08 | 0.009 | 13.55 | 24.22 | 1.79 |
| Ac-Orn | 0.26 | 0.05 | 0.26 | 0.24 | 0.37 |  | 0.16 | 0.29 | 0.30 | 0.26 | 0.14 | 0.33 | 0.25 | 1.04 | 0.56 | 1.39 | 0.041 | 0.23 | 0.71 | 3.06 |
| C5-OH (C3-DC-M) | 0.02 | 0.01 | 0.02 | 0.02 | 0.02 | 0.01 | 0.01 | 0.02 |  | 0.02 | 0.02 | 0.02 | 0.02 | 0.03 | 0.02 | 0.03 | 0.043 | 0.02 | 0.02 | 1.46 |
| Cit | 1.81 | 2.38 | 2.28 | 2.77 | 3.50 |  | 0.77 | 3.17 | 3.98 | 2.52 | 3.13 | 2.47 | 3.51 | 4.52 | 5.95 | 5.75 | 0.047 | 2.63 | 4.44 | 1.69 |


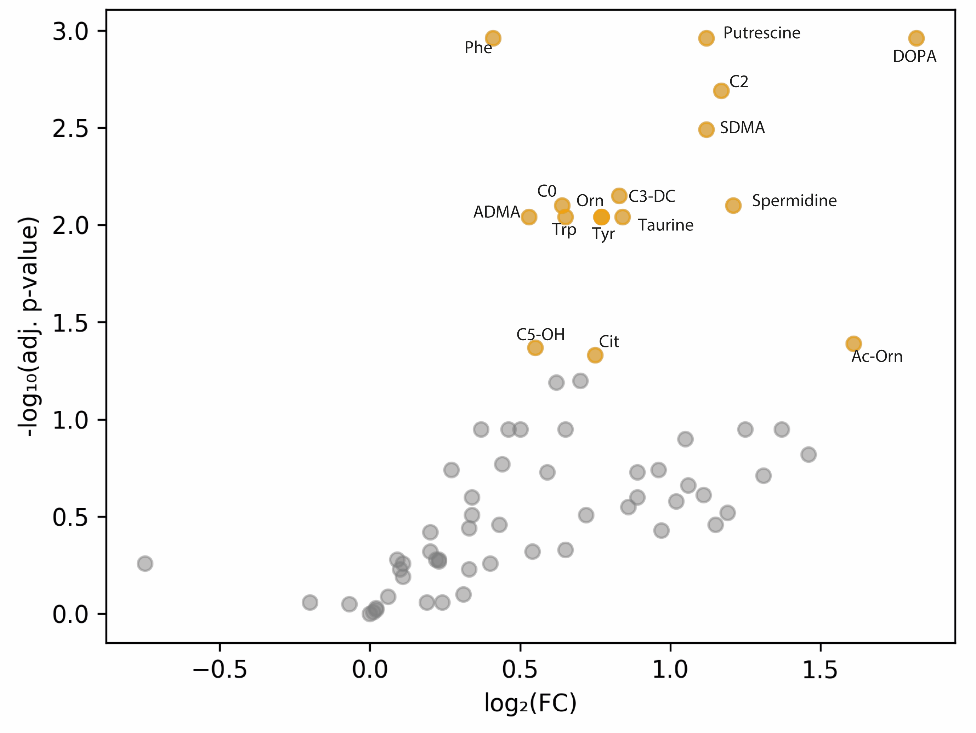


**Supplementary Figure S1. Volcano plot (statistical significance versus log_2_ fold change -FC-) upon comparing data from control and PD groups.** Virtually all metabolites were increased in the AH of PD patients. Metabolites whose concentration is significantly altered in the AH of patients with an adjusted p-value < 0.05 are indicated in orange. FC: Fold change.


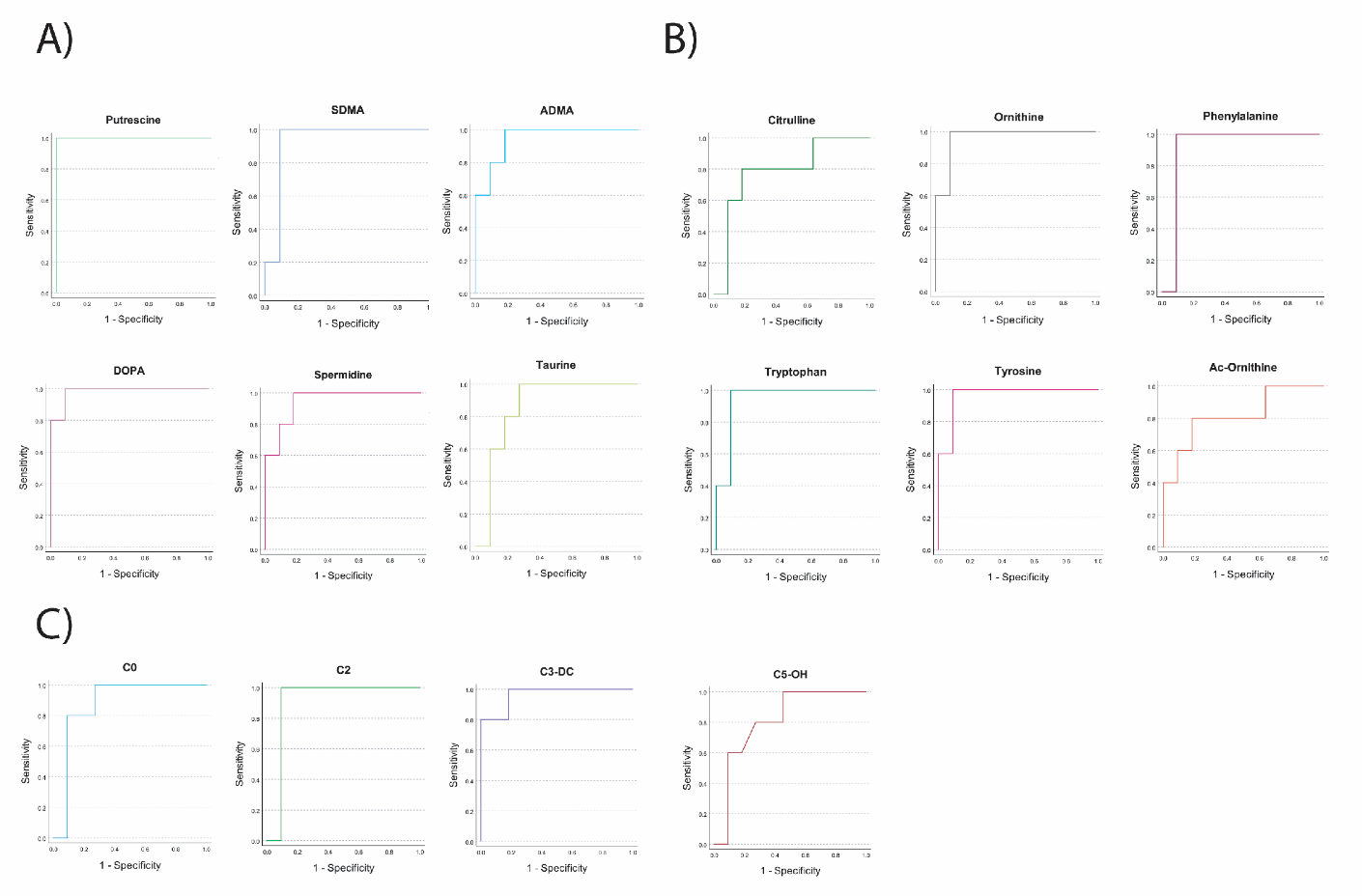


**Supplementary Figure S2. Receiver operating characteristic (ROC) curves for compounds in Figure 1 of the main paper. A.** Biogenic amines**. B.** Amino acids**. C.** Acylcarnitines.
